# The Effect of Curcumin Differs on Individual Cognitive Domains across Different Patient Populations: A Systematic Review and Meta-Analysis

**DOI:** 10.3390/ph14121235

**Published:** 2021-11-28

**Authors:** I-Chen Tsai, Chih-Wei Hsu, Chun-Hung Chang, Ping-Tao Tseng, Ke-Vin Chang

**Affiliations:** 1 Institute of Clinical Medicine, National Yang Ming Chiao Tung University, Taipei 112304, Taiwan; d49624004@ym.edu.tw; 2 Congenital Heart Disease Study Group, Asian Society of Cardiovascular Imaging, Seoul 13572, Korea; 3 InnovaRad Inc., Taichung 407004, Taiwan; 4 Department of Psychiatry, Kaohsiung Chang Gung Memorial Hospital, Chang Gung University College of Medicine, Kaohsiung 83301, Taiwan; harwicacademia@gmail.com; 5 Department of Computer Science and Information Engineering, National Cheng Kung University, Tainan 70101, Taiwan; 6 Institute of Clinical Medical Science, China Medical University, Taichung 404333, Taiwan; chang763@gmail.com; 7 Department of Psychiatry & Brain Disease Research Center, China Medical University Hospital, Taichung 404332, Taiwan; 8 An Nan Hospital, China Medical University, Tainan 709204, Taiwan; 9 Prospect Clinic for Otorhinolaryngology & Neurology, Kaohsiung 811022, Taiwan; ducktseng@gmail.com; 10 Institute of Biomedical Sciences, National Sun Yat-sen University, Kaohsiung 804201, Taiwan; 11 Department of Psychology, College of Medical and Health Science, Asia University, Taichung 41354, Taiwan; 12 Department of Physical Medicine and Rehabilitation, National Taiwan University Hospital, National Taiwan University College of Medicine, Taipei 10048, Taiwan; 13 Department of Physical Medicine and Rehabilitation, National Taiwan University Hospital, Bei-Hu Branch, Taipei 10845, Taiwan; 14 Center for Regional Anesthesia and Pain Medicine, Wang-Fang Hospital, Taipei Medical University, Taipei 11600, Taiwan

**Keywords:** cognition, cognitive function, *Curcuma longa*, curcumin, turmeric

## Abstract

Curcumin is a polyphenol with strong antioxidant and anti-inflammatory effects that has been shown to be effective in ameliorating cognitive decline in animal studies. However, its clinical effectiveness is inconclusive, and relevant gastrointestinal adverse events (AEs) have been reported. The aim of this meta-analysis was to summarize the existing evidence from randomized controlled trials (RCTs) of effects of curcumin on overall cognitive function, individual cognitive domains, and gastrointestinal AE. The study includes 8 RCTs and 389 participants. A random-effects model was used for the meta-analysis. Compared with the placebo group, the curcumin group was associated with an improvement in working memory (Hedges’ *g* = 0.396, 95% confidence interval (CI) = 0.078 to 0.714, *p* = 0.015) and a borderline benefit in processing speed (Hedges’ *g* = 0.303, 95% CI = −0.013 to 0.619, *p* = 0.06). In the domains of language, episodic memory/visual learning, verbal memory, cognitive flexibility/problem solving, and overall cognitive function, no significant difference existed for the comparison between the curcumin and placebo groups. The curcumin group had a significantly higher risk of gastrointestinal AEs than the placebo group (odds ratio = 3.019, 95% CI = 1.118 to 8.150, *p* = 0.029). In the future, the effects of curcumin on working memory, processing speed, and gastrointestinal AE should be further investigated.

## 1. Introduction

### 1.1. Curcumin in Cognitive Decline Animal Studies

Curcumin is a polyphenol with strong antioxidant and anti-inflammatory activity [1,2,3]. It is also the primary active component of the Indian spice turmeric (*Curcuma longa*) [4] and curry powders [5]. Animal studies have also confirmed that curcumin has protective effects against cognitive decline, both in aging rhesus monkeys [6] and in a streptozotocin-induced dementia rat model [7]. Specifically, curcumin has been found to exhibit various selective effects, including preventing atrophic change and cell loss in the prefrontal cortex compared to the hippocampus [7,8], decreasing neuroinflammation compared to promoting neurogenesis [7], and more positive effects on working memory than on recognition [6].

### 1.2. Curcumin in Cognitive Epidemiological Studies and Clinical Trials

A multi-ethnic Asian study found that, in older adults aged 60 years or more, those who consumed curry “occasionally”, “often”, or “very often” had significantly better Mini-Mental State Examination (MMSE) scores than subjects who “never” or “rarely” consumed curry [9]. However, the results of randomized controlled trials (RCTs) are controversial. For example, Ringman et al. found that the curcumin group did not show clinical or biochemical evidence of efficacy in cognitive function tests and biomarkers in patients with Alzheimer’s disease [10]. In contrast, Rainey-Smith et al. found that the curcumin group maintained cognitive function during the study period, while the placebo group kept deteriorating [11].

Zhu et al. published a meta-analysis investigating the effect of curcumin on cognition in 2019 [12], summarizing the RCTs available between 2008 and 2018 [10,11,13,14,15,16]. They also performed a subgroup analysis according to participants based on the presence of older age, Alzheimer’s disease, and schizophrenia. They found that curcumin might be beneficial for the overall cognition of healthy and non-demented older adults. However, in Alzheimer’s disease, curcumin seems to have a detrimental effect compared with placebo (standardized mean difference = −0.9, *p* = 0.002). Since Alzheimer’s disease tends to involve the prefrontal cortex [8] and cause corresponding working memory deficits [17], these differences in outcomes across healthy and diseased populations may indicate the selective effects of curcumin on different brain regions and cognitive domains.

### 1.3. Individual Cognitive Domains and Gastrointestinal Adverse Events

The most important age-related changes occur in the domains of working memory, processing speed, and problem-solving (executive function) [18]. Alzheimer’s disease is characterized by a decline in working memory, episodic memory, and problem solving [19]. Schizophrenia is known for its multi-domain involvement, including working memory, verbal memory, attention, and executive function [20]. Since earlier human studies usually treat cognitive function as a whole [3,12], the effects of curcumin on different cognitive domains should also be further investigated.

Although curcumin is a safe natural compound that can be ingested up to 6 g/day for 4–7 weeks [21] without reported toxicity, side effects, such as gastrointestinal upset and diarrhea, have been observed [21,22,23]. The aim of this study was to review the available RCTs with their corresponding data in a trial registry to provide an updated meta-analysis on the effect of curcumin on overall cognitive function and individual cognitive domains. In this meta-analysis, we also analyzed the side effects and pertinent withdrawal rates of each included trial.

## 2. Results

### 2.1. Study Selection

**Figure 1:**
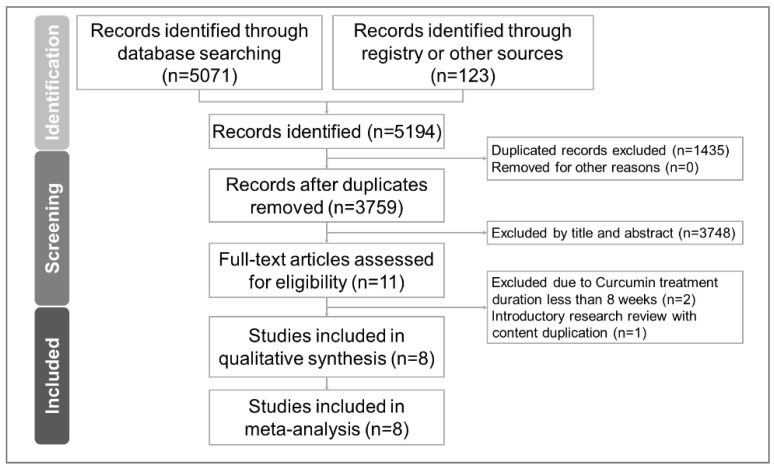
PRISMA 2020 flowchart of current meta-analysis.

The PRISMA flow chart of the literature search process is presented in Figure 1. After removing the duplicated articles and excluding non-relevant articles by reading the titles and abstracts, we found 11 RCTs evaluating the effect of curcumin on cognitive function. Two articles were excluded due to an intervention duration of less than 8 weeks [14,24]. One article was excluded because it was a review of an already-included RCT [25] (Table S3). Finally, eight articles were included [10,11,13,15,16,26,27,28]. The clinical trial registry data of these eight articles were also checked and analyzed for qualitative and quantitative analyses [29,30,31,32,33,34,35]. In this meta-analysis, all the data needed were successfully retrieved from published articles, accompanying Supplementary Materials, and clinical trial registries.

In these eight eligible RCTs, there were 389 participants with an age of 65.0 (mean) ± 10.2 (standard deviation) years and 44.7% (n = 174) males. All studies were double-blinded. The study duration ranged from 8 to 18 months. The enrolled subjects included older adults in four studies [11,15,27,28], patients with Alzheimer’s disease in two studies [10,13], and patients with schizophrenia in two studies [16,26]. The curcumin formulations were Theracumin^®^ in three studies [15,16,26], Brain Active^®^ in one study [28], Longvida^®^ in one study [27], Biocurcumax^®^ in one study [11], Curcumin C3 Complex^®^ in one study [10], and powders of Kancor flavors or capsules of Arjuna Natural Extracts in one study [13]. The daily dose of curcumin ranged from 80 to 4000 mg.

The curcumin dosage, study arms, and cognitive function test results are summarized in Table 1. For studies with more than one test in a specific cognitive domain, the most representative test was selected by consensus and listed in the first line of the corresponding cells. For example, in Cox et al.’s study published in 2020 [27], there were serial seven subtraction, serial three subtraction, and virtual Morris water maze (vMWM) tests in the working memory domain. Serial seven was selected as the representative test because it had the best differentiating power [17,36]. In another example, in Ringman et al.’s study published in 2012 [10], ADAS-Cog and MMSE were used to assess overall cognitive function. We selected ADAS-Cog because it is more sensitive and detailed than the MMSE (Table 1) [37].

### 2.2. Methodological Quality of Included Studies

**Figure 2:**
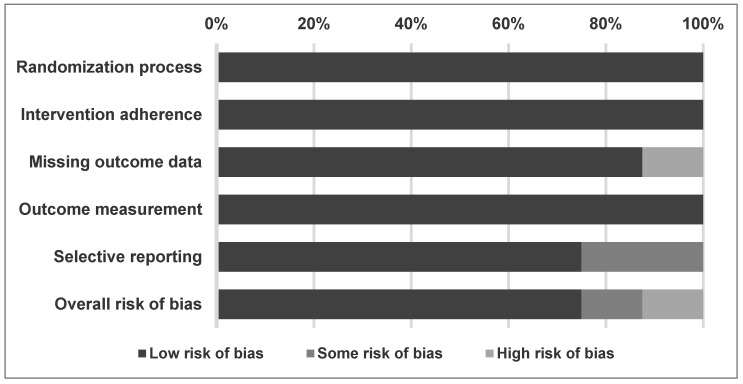
Summary of quality assessment of studies included in the meta-analysis using Cochrane risk of bias 2 tool.

Regarding the overall methodological quality of the studies included in the meta-analysis, 75% of the studies had low risk of bias, 12.5% had some risk of bias, and 12.5% had a high risk of bias (Figure 2). In a detailed assessment, one study was rated as “high” risk of bias in missing outcome data [11] and two studies were rated as “some” risk of bias in selective reporting [11,15]. Notably, we rated Wynn et al.’s study [16] as “low” risk of bias in all aspects, which was different from the assessment from Zhu et al.’s meta-analysis [39] in the domain of incomplete outcome data and selective reporting [12]. This was because Wynn et al. disclosed the full results on ClinicalTrials.gov after Zhu et al.’s article was published [32]. The reasons for rating “some” and “high” risk of bias are listed in Table 2.

### 2.3. Primary Outcome

#### 2.3.1. Overall Cognitive Function

**Figure 3:**
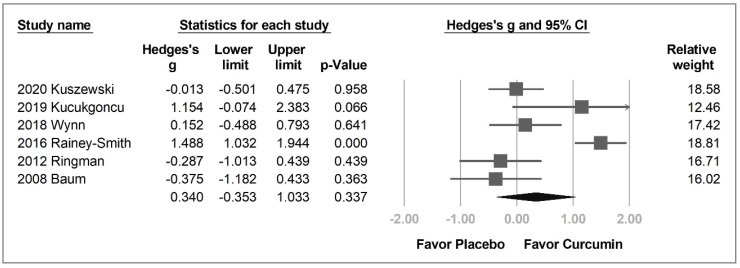
Forest plot of the effect of curcumin on the overall cognitive function.

Six out of the eight enrolled studies had data on overall cognitive assessment. No statistical difference (Hedges’ *g* = 0.340, 95% CI = −0.353 to 1.033, *p* = 0.337, *I*^2^ = 0.0%) was observed between the curcumin and placebo groups (Figure 3).

The participants were further grouped into older adults, patients with Alzheimer’s disease, and patients with schizophrenia for subgroup analysis. However, regardless of the group, curcumin was no more effective than placebo in overall cognitive function (older adults: Hedges’ *g* = 0.740, 95% CI = −0.731 to 2.211, *p* = 0.324, *I*^2^ = 94.9%; Alzheimer’s disease: Hedges’ *g* = −0.326, 95% CI = −0.866 to 0.214, *p* = 0.237, *I*^2^ = 0.0%; schizophrenia: Hedges’ *g* = 0.511, 95% CI = −0.431 to 1.452, *p* = 0.288, *I*^2^ = 50.2%) (Figure S1).

In the six studies analyzed, Rainey-Smith et al.’s [11] and Kucukgoncu’s [26] studies were the only two with ES more than 1.0, and were considered to be the source of heterogeneity.

#### 2.3.2. Individual Cognitive Domains

**Figure 4:**
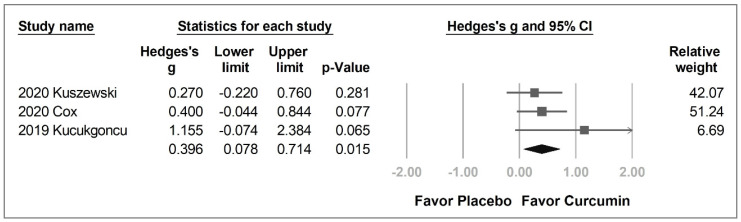
Forest plot of the effect of curcumin on working memory.

With regards to the individual domains, working memory was the only domain with significant between-group differences. From the data of the three enrolled studies [26,27,28], the curcumin group significantly improved working memory compared with the placebo group (Hedges’ *g* = 0.396, 95% CI = 0.078 to 0.714, *p* = 0.015) without significant heterogeneity (*I*^2^ = 0.0%) (Figure 4).

Sensitivity analysis was also performed. In working memory, we selected a serial seven subtraction test to represent Cox et al.’s study [27], rather than serial three subtraction or vMWM tests. To confirm that the result was not affected by the decision, we used serial three subtraction and vMWM tests instead. The substitutions did not alter the statistical significance (serial three subtraction: Hedges’ *g* = 0.333, 95% CI = 0.016 to 0.650, *p* = 0.039, *I*^2^ = 0.0%; vMWM: Hedges’ *g* = 0.454, 95% CI = 0.135 to 0.773, *p* = 0.005, *I*^2^ = 0.0%) (Figures S2 and S3).

**Figure 5:**
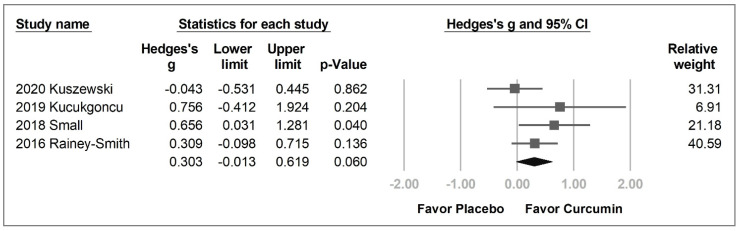
Forest plot of effect of curcumin on processing speed.

The processing speed in the curcumin group was also likely to be better than that in the placebo group with a borderline *p* value (Hedges’ *g* = 0.303, 95% CI = −0.013 to 0.619, *p* = 0.060, *I*^2^ = 19.5%) (Figure 5).

There was no statistical difference between the curcumin and placebo groups in the domains of language (Hedges’ *g* = −0.633, 95% CI = −1.878 to 0.612, *p* = 0.319, *I*^2^ = 93.0%; Figure S4), episodic memory/visual learning (Hedges’ *g* = −0.075, 95% CI = −0.438 to 0.287, *p* = 0.683, *I*^2^ = 0.0%; Figure S5), verbal memory (Hedges’ *g* = 0.273, 95% CI = −0.190 to 0.739, *p* = 0.248, *I*^2^ = 22.3%; Figure S6), and cognitive flexibility/problem solving (Hedges’ *g* = 0.050, 95% CI = −0.482 to 0.583, *p* = 0.853, *I*^2^ = 44.4%; Figure S7).

### 2.4. Secondary Outcomes

**Figure 6:**
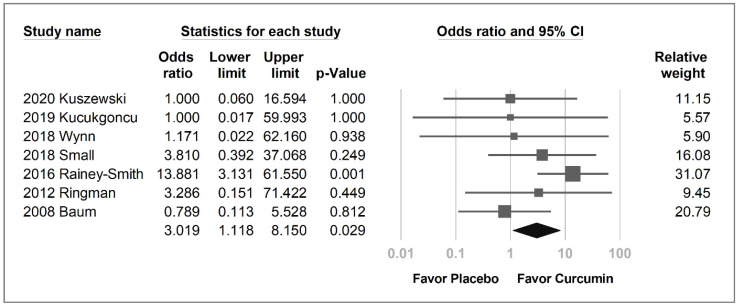
Forest plot of the gastrointestinal adverse event rate of curcumin compared with the placebo group.

The curcumin group was likely to have a higher overall withdrawal rate than the placebo group, with a borderline *p*-value (OR = 1.643, 95% CI = 0.980–2.753, *p* = 0.059, *I*^2^ = 5.5%; Figure S8). The adverse event-related withdrawal rate and adverse event rates were not significantly higher in the curcumin group, with an OR of 2.44 (95% CI = 0.791–6.361, *p* = 0.128, *I*^2^ = 45.1%) and 2.245 (95% CI = 0.803 to 6.276, *p* = 0.123, *I*^2^ = 46.5%), respectively (Figures S9 and S10). However, curcumin was associated with significantly more gastrointestinal adverse events than the placebo (OR = 3.019, 95% CI, 1.118–8.150, *p* = 0.029; *I*^2^ = 13.3%; Figure 6).

### 2.5. Publication Bias

No publication bias was detected in any funnel plot under visual inspection (Figures S11–S20).

## 3. Discussion

### 3.1. Main Results

Our meta-analysis found that curcumin supplementation and placebo regimens did not enhance overall cognitive function. However, curcumin improved working memory performance more than the placebo. This finding was validated by selecting different working memory tests for sensitivity analysis. Curcumin was also likely to be beneficial for processing speed. Finally, a higher rate of gastrointestinal adverse events was observed in the curcumin group than in the placebo group.

### 3.2. Possible Mechanisms of the Improved Cognitive Function by Curcumin

There are multiple pathways related to the cognitive function improvement after the use of curcumin. Anti-inflammation is one of them, since there were studies showing that cognitive declines in schizophrenia and mood disorders were associated with inflammation [40,41]. Curcumin can reduce inflammation and provide neuroprotection by inhibiting NFκB and subsequent TNF-α to reduce reactive oxygen species activation [42]. Furthermore, activation of the pathways of phosphatidylinositol-3/protein kinase B/glycogen synthase kinase-3 and phosphatidylinositol-3/protein kinase B/cAMP response element-binding protein/brain-derived neurotrophic factor were also found to reduce neurodegeneration [43].

### 3.3. Evidence Summary of Multisystem Health Benefits of Curcumin

Regarding the cardiovascular system, Ashtary-Larky et al. summarized nine RCTs and concluded that curcumin may reduce cardiovascular disease risk by improving glycemic and lipid profiles, scaling down inflammation, and reducing systolic blood pressure [44]. With reference to inflammatory diseases, such as ulcerative colitis, a meta-analysis of seven RCTs by Chandan et al. found that curcumin combined with mesalamine was associated with threefold better odds of a clinical response than mesalamine with placebo [45]. For physically active people and athletes, curcumin improved performance by reducing exercise-induced muscle damage and modulating the inflammation caused by physical activity, according to the systematic reviews of Suhett et al.’s and Fernández-Lázaro et al. [46,47]. To enhance the effects of cancer therapy, Mansouri et al. systematically reviewed 22 studies, concluding that curcumin could reduce the side effects of chemotherapy or radiotherapy and improve patients’ quality of life [48].

Zhu et al. synthesized six RCTs and found that curcumin and placebo did not differ with respect to overall cognitive function. However, Zhu et al. did not investigate the effects of curcumin on different cognitive domains due to the limited number of available studies at that time. Our meta-analysis further divided the cognitive tests into different domains and found that curcumin could improve working memory and possibly processing speed.

These multi-system health benefits are related to antioxidant and anti-inflammatory activities and may also be synergistic [49]. For example, the positive cognitive effects might be related not only to the direct effects of curcumin on neural cells [50], but also to its action on the cardiovascular system, such as protecting the endothelium, maintaining microvascular function, stabilizing cerebrovascular perfusion, and keeping the brain–blood barrier intact [51,52], which can also ameliorate neurodegenerative changes [53].

### 3.4. Effects of Curcumin on Different Cognitive Domains

The reason why working memory and processing speed improved more than other domains after the administration of the curcumin supplement may be related to its selective effect on different regions and cells.

Working memory is a unique, short-term active storage mechanism used to accomplish various cognitive activities [17]. Delayed-response tasks are usually used in animal studies to explore the relationship between working memory function and the underlying neural mechanism [17]. The studies of Watanabe et al. for monkey’s neural activity found that different working memory tasks activated overlapping prefrontal neural populations, and the magnitude of activity was related to the difficulty of the task [54,55]. The aforementioned findings indicate that working memory is mostly processed in the prefrontal cortex.

The protective effect of curcumin in the prefrontal cortex has also been demonstrated in animal models. Noorafshan et al. used three different neural damage rat models, including stress-induced, sulfite-induced, and sleep deprivation, and found that curcumin could prevent structural deterioration in neurons and glial cells, and could also counteract behavioral changes [8,56,57]. This showed that curcumin had a direct protective effect on the prefrontal cortex and may explain why curcumin was able to effectively improve working memory, as observed in our meta-analysis.

With regards to processing speed, the quantitative neuroimaging study of Magistro et al. on healthy young people found that the speed of processing was correlated with the volume of the white matter in the whole brain [58]. During the process of aging, a decreased overall processing speed and an increased variability of intra-individual processing speed has been observed in the elderly [59]. This decline in processing speed has been found to be accompanied by white matter changes on histopathology and neuroimaging [60,61]. The current cortical disconnection theory hypothesizes that a decline in white matter integrity disrupts the information flow within neural networks, which may be the cause of the reduction in processing speed [62,63].

Daverey et al. found that curcumin can inhibit hypoxia, inflammation, and apoptosis associated with white matter injury in rat cells [64]. Naeimi et al. used a rat model of demyelination and found that curcumin could significantly improve myelin repair and maintain white matter integrity [50]. These mechanisms may explain the beneficial effects of curcumin on the processing speed.

In contrast, using a literature search from the electronic database, we did not identify any animal or human studies demonstrating that curcumin could act on Broca’s area for language, occipital lobe for visual learning, fronto-temporo-parietal region for verbal memory, and the interaction between the thalamus and prefrontal cortex for cognitive flexibility. This may explain the non-significant results of curcumin for these cognitive domains.

### 3.5. Adverse Gastrointestinal Effects of Curcumin

Curcumin is categorized as “generally considered as safe” by United States Food and Drug Agency [65]. However, high-dose curcumin directly stimulates the gastrointestinal mucosa. In an extensive report published by the National Toxicology Program (United States), a two-year feeding study found increased incidences of ulcers, hyperplasia, and inflammation of the forestomach, cecum, and colon in male rats and of the cecum in female rats administered curcumin supplement [66]. A phase I clinical trial of oral curcumin included 15 patients, of whom one participant showed nausea and two participants experienced diarrhea, both of whose conditions could be medically controlled or spontaneously resolved [23].

The side effects of gastrointestinal irritation caused by curcumin may be dose-dependent. As experimental studies showed at the cellular level, low-concentration curcumin demonstrated antioxidant effects; however, at higher concentrations, it increased the intracellular reactive oxygen species [67,68,69]. When the concentration of curcumin in the cerebrospinal fluid reaches the threshold of cognitive benefit, the focal high concentration of curcumin in gastrointestinal mucosa cause inflammation and ulcers, with symptoms including nausea and diarrhea, similar to the observations in rat and mouse studies [66] and a phase I clinical trial [23].

In our study, we also found higher odds of gastrointestinal AEs in the curcumin group. We speculate that in the future, the trials of curcumin, including nutraceuticals designed for higher curcumin bioavailability [42], should emphasize adverse event reporting, and may consider an intention-to-treat study design to better preserve the prognostic balance of randomization [70].

### 3.6. Different Formulations and Ingredients

In the included trials, six different formulations of curcumin were used [71,72,73,74,75,76]. The core ingredients were produced by five different corporations [72,73,74,75,76]. The different formulations were all developed to enhance the bioavailability of curcumin. For example, Biocurcumax is made of curcumin with turmeric essential oil [73]. Theracumin is made of the submicron curcumin particles with surface preparation for preventing recrystallization [76]. Longvida uses solid lipid curcumin particles to bypass initial hydrolysis and to deliver unglucuronidated (free) curcumin to brain and other target tissues [75]. However, there was no systematic comparison of these different formulations regarding their clinical effectiveness on cognitive function.

In the included eight trials, five used curcumin and three used curcuminoids, which included curcumin, demethoxycurcumin, and bisdemethoxycurcumin. Though these three compounds showed different mechanisms of morphine’s rewarding effect in rat brain study [77], curcumin is considered to be the most potent [78,79] and is also the major ingredient (about 75–78%) in curcuminoids. The results implied that in our meta-analysis, the cognitive improvement and gastro-intestinal adverse events are likely to stem from the effect of curcumin.

### 3.7. Limitations

There are several limitations in our study. First, this meta-analysis synthesized different populations of participants, including older adults, and Alzheimer’s disease and schizophrenia patients. The differences in the underlying conditions might be associated with different cognitive changes under curcumin intervention. We performed a subgroup analysis to specifically address this issue, and found no significant difference among the three participant groups (Figure S1). Since the inflammation plays an important role in normal aging brain [80], Alzheimer’s disease [81], and schizophrenia [41], the anti-inflammatory effects of curcumin seemed similar in the three aforementioned groups. Furthermore, a transdiagnostic meta-analysis is a common and acceptable practice if the potential heterogeneity is well-examined. For example, Zhu et al. included older adults, Alzheimer’s disease and schizophrenia patients to evaluate the effect of curcumin in cognitive function [12], and Dauwan et al. included six different brain disorders to examine the effects of physical exercise on quality of life, depressive symptoms, and cognition [82].

Second, the number of eligible RCTs was small, resulting in only two to six datasets being extracted in each cognitive domain. Nevertheless, the significant benefit of curcumin in the working memory domain remained following the sensitivity analysis, indicating that the result was robust and not likely to be caused by the subjective decision of the index test.

Lastly, the RCTs in this field mostly employed the per-protocol design, which may inherently overestimate the treatment effect and underestimate the adverse-event-related dropout [83]. In this meta-analysis, we also used a per-protocol analysis to prevent distortion of the extracted data. In the future, for those who are designing a new RCT in this field, an intention-to-treat design is suggested to truly reflect the clinical effectiveness of curcumin.

## 4. Materials and Methods

### 4.1. General Guidelines

We followed the instructions of the latest version of PRISMA 2020 guidelines (Table S1) [84] and AMSTAR 2 (Assessing the Methodological Quality of Systematic Reviews) guidelines [85]. This study was registered in https://inplasy.com/inplasy-2021-9-0085/ (accessed on 24 September 2021, registered number: INPLASY202190085) [86].

### 4.2. Database Searches and Identification of Eligible Papers

Two authors (I.-C.T. and K.-V.C.) made independent electronic searches in the PubMed, Embase, ClinicalKey, Cochrane CENTRAL, ProQuest, ScienceDirect, and Web of Science with keyword of (“curcumin” OR “turmeric” OR “ginger” OR “curcuma longa” OR “curcuminoid”) AND (“cognitive” OR “cognition” OR “memory” OR “learning” OR “dementia” OR “Alzheimer”) through the earliest record up to 18 August 2021. In addition, to include gray literature and unpublished data, we also performed an electronic search on the ClinicalTrials.gov platform. A detailed search strategy is provided in the Supplementary Material (Table S2).

In the initial stage, these two authors screened the titles and abstracts for eligibility through consensus. To expand the pool of potential studies, we scrutinized the reference lists of review articles [3,12,38,87] and performed further manual searches. Later, a third reviewer (P.-T.T.) was consulted in situations in which the two authors could not achieve consensus. No language restrictions were imposed.

### 4.3. Inclusion and Exclusion Criteria

The PICO (population, intervention, comparison, and outcome) setting of the current meta-analysis included: (1) P: human adult participants (≥20 years old); (2) I: curcumin oral supplement; (3) C: placebo; and (4) O: changes in the scores of the selected cognitive function tests.

To generate a recruited study list, the following inclusion criteria were used: (1) RCTs with adult human participants (≥20 years old), (2) RCTs investigating the difference in results of cognitive function tests after curcumin supplementation or placebo regimens, (3) RCTs with an intervention duration greater than or equal to 8 weeks, and (4) placebo-controlled trials. To be specific, we chose the least treatment duration of 8 weeks, which is the recommended length of curcumin supplement to take effect [88,89].

The exclusion criteria were as follows: (1) studies that were not RCTs; (2) studies that included participants <20 years old; (3) studies that did not investigate the influence of curcumin supplement and placebo regimens on cognition; (4) an introductory research review that simply reports previous studies; (5) studies with an intervention duration of less than 8 weeks; (6) studies that lacked placebo or controlled arms.

### 4.4. Methodological Quality Appraisal

To investigate the methodological quality of the recruited studies, we used version 2 of the Cochrane risk-of-bias tool for randomized trials (RoB 2), which consists of six main items: randomization process, intervention adherence, missing outcome data, outcome measurement, selective reporting, and overall risk of bias [83].

In RoB 2′s intervention adherence section, one of the two options should be selected for literature assessment: intention-to-treat (intervention assignment) or per-protocol (intervention adherence). In this meta-analysis, we chose a per-protocol evaluation [83]. Any discrepancy in the opinions of assessments between the two evaluators (I.-C.T. and K.-V.C.) was solved by the judgment of the third evaluator (P.-T.T.).

### 4.5. Primary Outcomes (Changes in Cognitive Function)

The primary outcomes were changes in cognitive function tests following at least 8-weeks of use of curcumin or placebo regimens. Cognitive function tests were categorized into nine domains, including overall performance, working memory, processing speed, language, episodic memory/visual learning, verbal memory, cognitive flexibility/problem solving, social cognition, and fluid cognition [90].

### 4.6. Secondary Outcomes (Withdrawal Rates and Adverse Event Rates)

The secondary outcomes were: (1) overall withdrawal rates; (2) specific withdrawal rates related to adverse effects; (3) overall adverse event rates; (4) gastrointestinal adverse event rates. For cells with zero-event, the zero was replaced by 0.5 to enable software calculation to properly include the study in to the analysis [91]. The aforementioned outcomes were quantified and synthesized using odds ratios (ORs).

### 4.7. Data Extraction and Management

Two independent authors (I.-C.T. and K.-V.C.) extracted data from the recruited studies, including demographic data, study design, details of curcumin supplement and placebo regimens, and the primary and secondary outcomes. In situations where the data were unavailable in the published articles and clinical trial registries, we contacted the corresponding authors to request the original data.

The combination of the results from the arms using different doses of curcumin was processed according to suggestions from the Cochrane Handbook for Systematic Reviews of Interventions [92]. For studies with multiple treatment arms, those without the use of curcumin or placebo regimens were not included in the quantitative analysis [93].

### 4.8. Statistical Analysis

Based on the heterogeneous target populations in the recruited studies, the meta-analysis was conducted using a random-effects model [94]. The meta-analysis was performed using the Comprehensive Meta-Analysis software, version 3 (Biostat, Englewood, NJ, USA). We chose Hedges’ *g* and 95% confidence intervals (95% CI) as the main effect size (ES) of the primary outcomes (cognitive function changes in different domains). A Hedges’ *g* of 0.2, 0.5, and 0.8, was considered a small, moderate, and large ES, respectively [95]. We chose ORs and their 95% CIs to investigate secondary outcomes (rates of withdrawal and adverse events). Subgroup analysis was performed based on the differences in the target populations, such as older adults, Alzheimer’s disease, and schizophrenia. *I*^2^ and Cochran’s *Q* statistics were used to evaluate the degree of heterogeneity among the studies. *I*^2^ values of 25%, 50%, and 75% were considered low, moderate, and high heterogeneity, respectively [96].

If there was more than one test in a specific domain, the test that best discriminated the outcome change was selected for meta-analysis inclusion by consensus of two authors (I.-C.T. and K.-V.C.). For example, serial 7 and serial 3 subtraction tests were used for the evaluation of working memory [36]. Sensitivity analysis was also performed by substituting the representative test with other similar assessments and re-analyzed to check whether the association between the intervention and outcomes changed significantly [91]. Potential publication bias was evaluated according to the Cochrane Handbook for Systematic Reviews of Interventions [97]. We visually inspected funnel plots when there were fewer than 10 datasets. Egger’s regression tests were performed when there were 10 or more datasets. A two-tailed *p*-value less than 0.05 was considered statistically significant.

## 5. Conclusions

Following at least 8 weeks of nutritional supplementation, curcumin was found to improve working memory more than a placebo regimen. The results in the cognitive domain of processing speed were notable, with a borderline *p*-value, which may be significant if further trials are included. Moreover, curcumin was associated with higher odds of gastrointestinal AEs than the placebo regimen. Future RCTs should be designed and reported under the intention-to-treat principle to better reflect the true effectiveness of curcumin in the real world.

## Figures and Tables

**Table 1:** Summary of the retrieved trials investigating the effect of curcumin on cognitive functions.

Study	Kuszewski et al.	Cox et al.	Kucukgoncu et al.	Wynn et al.	Small et al.	Rainey-Smith et al.	Ringman et al.	Baum et al.
Reference/ Registry (if available)	[35,38]	[27,34]	[26,33]	[16,32]	[15,31]	[11,30]	[10,29]	[13]
Year	2020	2020	2019	2018	2018	2016	2012	2008
Location	Australia	Australia	United States	United States	United States	Australia	United States	China
Funding/grant	Blackmores Institute *	Verdure Sciences *	The State of Connecticut	Not mentioned	NIH grants USDE contract Foundations Professorships	McCusker Alzheimer’s Research Foundation Hollywood Private Hospital Research Foundation	J. D. F. Alzheimer’s Disease Foundation Institute for the Study of Aging	CUHK Bupa Foundation
Design	RCT, double-blind	RCT, double-blind	RCT, double-blind	RCT, double-blind	RCT, double-blind	RCT, double-blind	RCT, double-blind	RCT, double-blind
Allocation conceal	Independent investigator	External staff	Not mentioned	Not mentioned	Research pharmacy	Not mentioned	Research pharmacy	Not mentioned
Randomization	Minimization method	Stratified	Not mentioned	Not mentioned	Randomization table	Not mentioned	Block randomization	Stratified
Study duration	16 weeks	12 weeks	8 weeks	8 weeks	18 months	12 months	24 weeks	6 months
Subjects	Overweight older adults	Healthy older adults	Schizophrenia	Schizophrenia	Non-demented older adults	Older adults	Alzheimer’s disease	Alzheimer’s disease
Curcumin product	Brain Active^®^ (Longvida^®^)	Longvida^®^	Theracumin^®^	Theracumin^®^	Theracumin^®^	Biocurcumax^®^	Curcumin C3 complex^®^	Powder or capsule
Curcumin manufacturer	Blackmores	Verdure Sciences	Theravalues	Theravalues	Theravalues	Arjuna Natural	Sabinsa	Kancor Flavors Arjuna Natural
Curcumin arms (N)	160 mg/d curcumin (31) Placebo (32)	80 mg/d curcumin (42) Placebo (43) (12 weeks: 39/40)	180 mg/d curcumin (6) Placebo (6) (8 weeks: 5/5)	360 mg/d curcumin (17) Placebo (19)	180 mg/d curcumin (21) Placebo (19)	1.32 g/d curcuminoids (39) Placebo (57)	2 g/d curcuminoids (10) 4 g/d curcuminoids (9) Placebo (11)	1 g/d curcuminoids (8) 4 g/d curcuminoids (11) Placebo (8)
Age (years)	160 mg/d: 65.7 ± 1.4 Placebo: 65.8 ± 1.4	80 mg/d: 67.8 ± 6.0 Placebo: 68.4 ± 6.7	41.3 ± 12.7	360 mg/d: 50.1 ± 9.6 Placebo: 50.9 ± 10.6	180 mg/d: 63.1 ± 8.4 Placebo: 62.9 ± 9.4	66 ± 6.6	2 g/d: 76.7 ± 5.6 4 g/d: 75.3 ± 6.9 Placebo: 70.2 ± 12.4	1 g/d: 69.0 ± 10.9 4 g/d: 73.4 ± 6.6 Placebo: 77.8 ± 7.7
Male %	160 mg/d: 48% Placebo: 44%	80 mg/d: 50% Placebo: 48.84%	Total: 75%	360 mg/d: 64.7% Placebo: 100%	180 mg/d: 43% Placebo: 47%	1.32 g/d: 33.3% Placebo: 26.3%	2 g/d: 33% 4 g/d: 30% Placebo: 45%	1 g/d: 12.5% 4 g/d: 27.3% Placebo: 37.5%
Cognition domains	NIH toolbox+	E-prime 2.0	MCCB	MCCB	Customized	Customized	ADAS-Cog & MMSE	MMSE
Overall	Overall performance	N/A	Composite score	MCCB T-score	N/A	MoCA Non-computerized and Computerized composite scores	ADAS-Cog MMSE	MMSE
Working memory	Working memory	Serial 7 subtraction Serial 3 subtraction vMWM	Working memory	N/A	N/A	N/A	N/A	N/A
Processing speed	Processing speed	N/A	Processing speed	N/A	Trail making test part A	Wechsler digit symbol scale	N/A	N/A
Language	Language	N/A	N/A	N/A	N/A	COWAT	N/A	N/A
Episodic memory /visual learning	Episodic memory	N/A	Visual learning	N/A	BVMT-R recall BVMT-R delay	N/A	N/A	N/A
Verbal memory	Verbal memory	DATT recognition accuracy DATT response time	Verbal learning	N/A	BSRT CLTR BSRT total BSRT long-term storage	RAVLT list A trial 1–5 total RAVLT short-term recall RAVLT delayed recall	N/A	N/A
Cognitive flexibility/problem solving	Cognitive flexibility	Arrow flankers test	Attention-vigilance Problem solving	N/A	N/A	N/A	N/A	N/A
Social cognition	N/A	N/A	Social cognition	N/A	N/A	N/A	N/A	N/A
Fluid cognition	Fluid cognition	N/A	N/A	N/A	N/A	N/A	N/A	N/A

* Manufacturer of the tested curcumin product. ADAS-Cog: Alzheimer’s Disease Assessment Scale-Cognitive Subscale, BSRT: Buschke Selective Reminding Test, BVMT-R: Brief Visual Memory Test-Revised, CLTR: Consistent Long Term Recall, COWAT: Controlled Oral Word Association Task, CUHK: The Chinese University of Hong Kong, DATT: Divided Attention Tracking Task, J.D.F: John Douglas French, MATRICS: Measurement and Treatment Research to Improve Cognition in Schizophrenia; MCCB: MATRICS™ Consensus Cognitive Battery; MoCA: Montreal Cognitive Assessment, N/A: not available, NIH: National Institute of Health, RCT: Randomized Controlled Trial, USDE: United States Department of Energy, vMWM: Virtual Morris Water Maze.

**Table 2:** Detailed quality assessment of included studies using Cochrane risk of bias 2 tool.

	Kuszewski et al.	Cox et al.	Kucukgoncu et al.	Wynn et al.	Small et al.	Rainey-Smith et al.	Ringman et al.	Baum et al.
Reference/ Registry (if available)	[35,38]	[27,34]	[26,33]	[16,32]	[15,31]	[11,30]	[10,29]	[13]
Year	2020	2020	2019	2018	2018	2016	2012	2008
Randomization process	L	L	L	L	L	L	L	L
Intervention adherence	L	L	L	L	L	L	L	L
Missing outcome data	L	L	L	L	L	H ^3^	L	L
Outcome measurement	L	L	L	L	L	L	L	L
Selective reporting	L	L	L	L ^1^	S ^2^	S ^4^	L	L
Overall RoB	L	L	L	L ^1^	S	H	L	L

H, high risk of bias; L, low risk of bias; RoB, risk of bias; S, risk of bias. ^1^ The assessment result is different from a previous meta-analysis conducted by Zhu et al. [12], because the authors then published results on ClinicalTrials.gov (accessed on 13 August 2021) and provided full results online. ^2^ The outcome was assumed to be reported at baseline and at the 18-month follow-up based on information from the trial registration website [31]. However, 6- and 12-month results were provided in the published article in addition to baseline and 18-month results [15]. Since we did not use the 6- and 12-month data for meta-analysis, some risk of bias was considered. ^3^ This study was determined to have a high risk of bias in missing outcomes because many adverse event-related withdrawals were noted, especially in the curcumin group (21/80) compared with the placebo group (2/80). ^4^ The trial was registered for baseline and 12-month evaluations [30]. However, 6-month results were provided in the published article in addition to the baseline and 12-month results [11]. Since we did not use the 6-month data for meta-analysis, some risk of bias was considered.

## Data Availability

Data is contained within the article and Supplementary Files.

## Supplementary Materials

The following are available online at https://www.mdpi.com/article/10.3390/ph14121235/s1, Figure S1: Subgroup analysis forest plot of the effect of curcumin on overall cognitive performance grouped by the participants’ characteristics, Figure S2: Forest plot of the effect of curcumin on working memory using serial 3 as the index test in Cox et al.’s study, Figure S3: Forest plot of the effect of curcumin on working memory using virtual Morris water maze (vMWM) task as the index text in Cox et al.’s study, Figure S4: Forest plot of the effect of curcumin on language, Figure S5: Forest plot of the effect of curcumin on episodic memory/visual learning, Figure S6: Forest plot of the effect of curcumin on verbal memory, Figure S7: Forest plot of the effect of curcumin on cognitive flexibility/problem solving, Figure S8: Forest plot of the effect of curcumin on withdrawal rate compared with the placebo, Figure S9: Forest plot of the effect of curcumin on adverse event-related withdrawal rate compared with the placebo, Figure S10: Forest plot of the effect of curcumin on adverse event rate compared with the placebo, Figure S11: Funnel plot of the studies evaluating overall cognitive performance, Figure S12: Funnel plot of the studies evaluating working memory, Figure S13: Funnel plot of the studies evaluating processing speed, Figure S14: Funnel plot of the studies evaluating episodic memory/visual learning, Figure S15: Funnel plot of the studies evaluating verbal memory, Figure S16: Funnel plot of the studies evaluating cognitive flexibility/problem solving, Figure S17: Funnel plot of the studies evaluating withdrawal rate, Figure S18: Funnel plot of the studies evaluating the adverse event-related withdrawal rate, Figure S19: Funnel plot of studies the evaluating the adverse event rate, Figure S20: Funnel plot of the studies evaluating the gastrointestinal adverse event rate, Table S1: Excluded studies and reasons, Table S2: PRISMA Checklist, Table S3: Keywords and search results in different databases.
