# Supplementary material for: The Effect of Curcumin Differs on Individual Cognitive Domains across Different Patient Populations: A Systematic Review and Meta-Analysis
Source: Pharmaceuticals (Basel). 2021 Nov 28;14(12):1235. doi: 10.3390/ph14121235 (PMC8708668; doi:10.3390/ph14121235)
Supplement: Supplementary file 1 [file pharmaceuticals-14-01235-s001.zip › pharmaceuticals-1471812-supplementary.pdf]

Supplementary Figures / Part 1 – Forest Plots

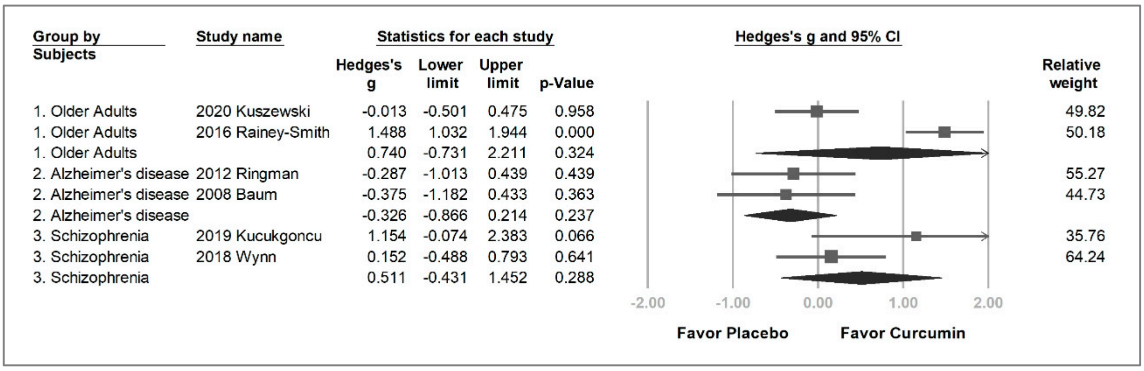

Figure S1. Subgroup analysis forest plot of the effect of curcumin on overall cognitive performance grouped by the participants' characteristics (older adults: Hedges' g = 0.740, 95% CI = –0.731 to 2.211, p = 0.324, I^2 = 94.9%; Alzheimer's disease: Hedges' g = –0.326, 95% CI = –0.866 to 0.214, p = 0.237, I^2 = 0.0%; Schizophrenia: Hedges' g = 0.511, 95% CI = –0.431 to 1.452, p = 0.288, I^2 = 50.2%)

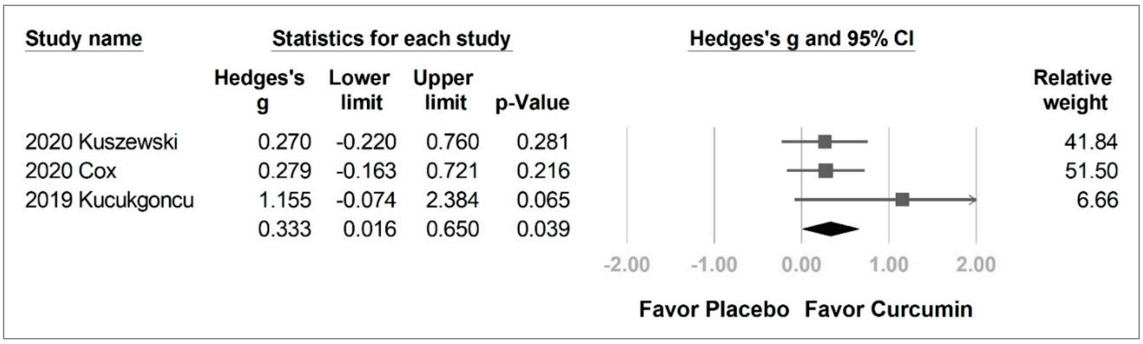

Figure S2. Forest plot of the effect of curcumin on working memory using serial 3 as the index test in Cox et al.'s study (serial 3 subtraction: Hedges' g = 0.333, 95% CIs = 0.016 to 0.650, p = 0.039, I^2 = 0.0%)

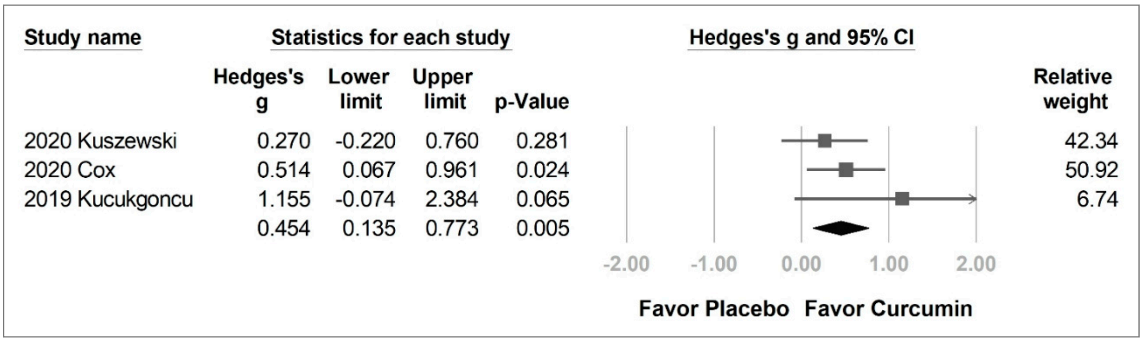

Figure S3. Forest plot of the effect of curcumin on working memory using virtual Morris water maze (vMWM) task as the index text in Cox et al.'s study (vMWM: Hedges' g = 0.454, 95% CI = 0.135 to 0.773, p = 0.005, I^2 = 0.0%)

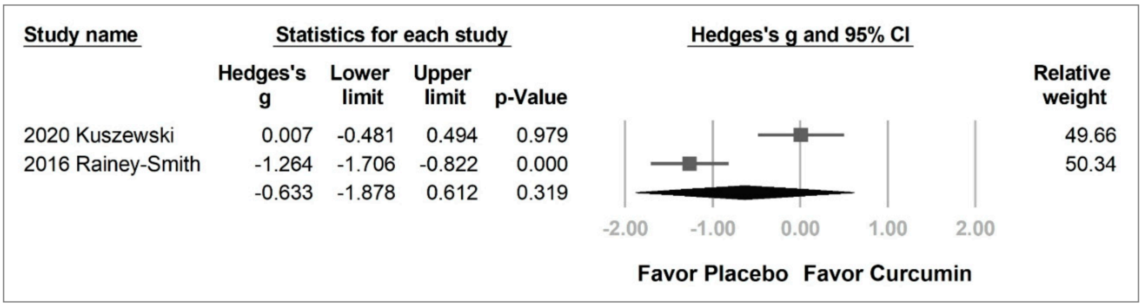

Figure S4. Forest plot of the effect of curcumin on language (Hedges' *g* = –0.633, 95% CI = –1.878 to 0.612, *p* = 0.319, I^2 = 93.0%)

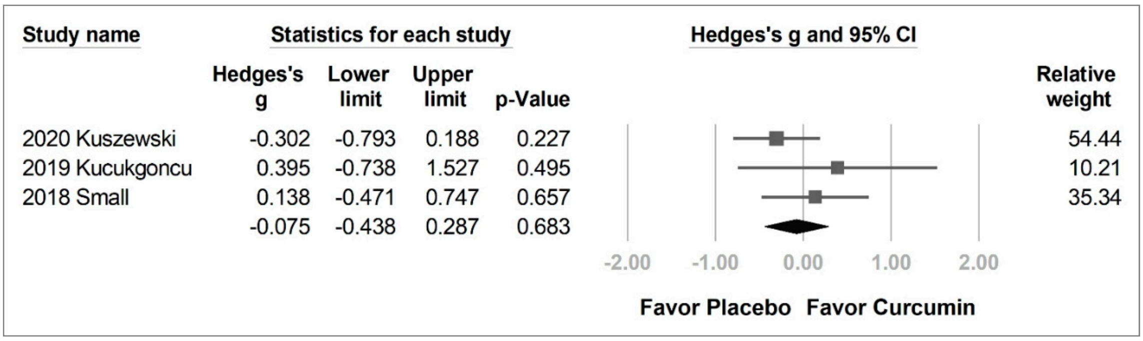

Figure S5. Forest plot of the effect of curcumin on episodic memory/visual learning (Hedges' *g* = –0.075, 95% CI = –0.438 to 0.287, *p* = 0.683, I^2 = 0.0%)

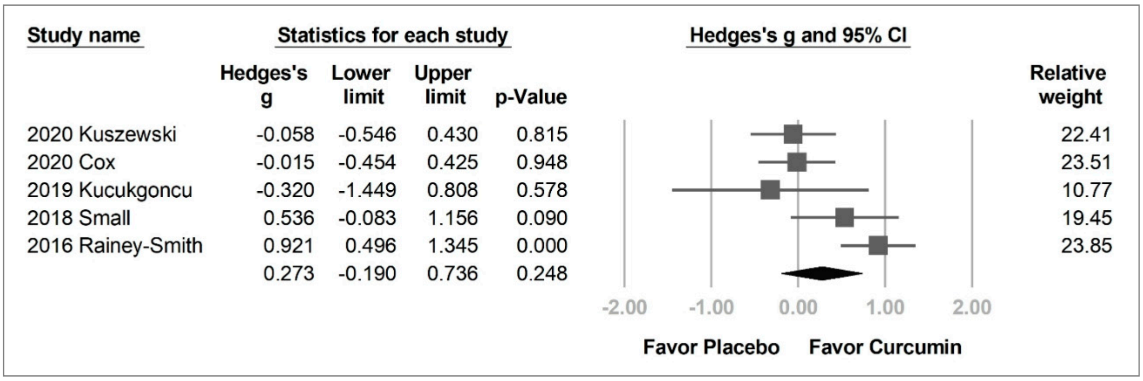

Figure S6. Forest plot of the effect of curcumin on verbal memory (Hedges' *g* = 0.273, 95% CI = –0.190 to 0.739, *p* = 0.248, I^2 = 22.3%)

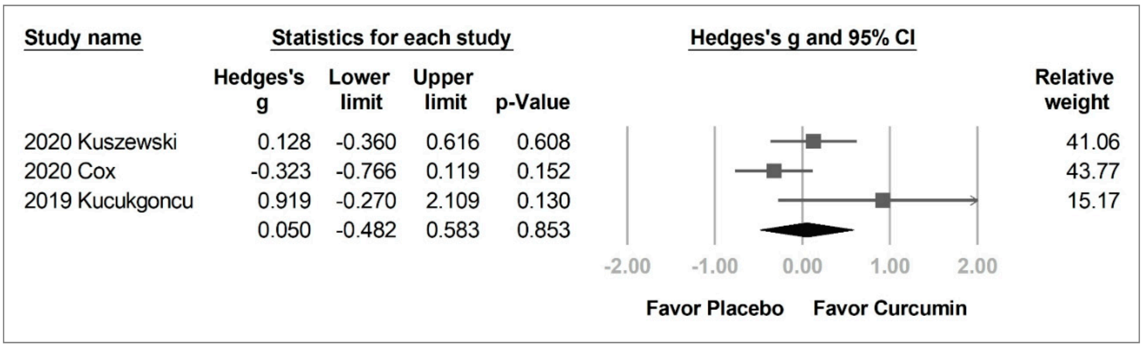

Figure S7. Forest plot of the effect of curcumin on cognitive flexibility/problem solving (Hedges' *g* = 0.050, 95% CI = –0.482 to 0.583, *p* = 0.853, I^2 = 44.4%)

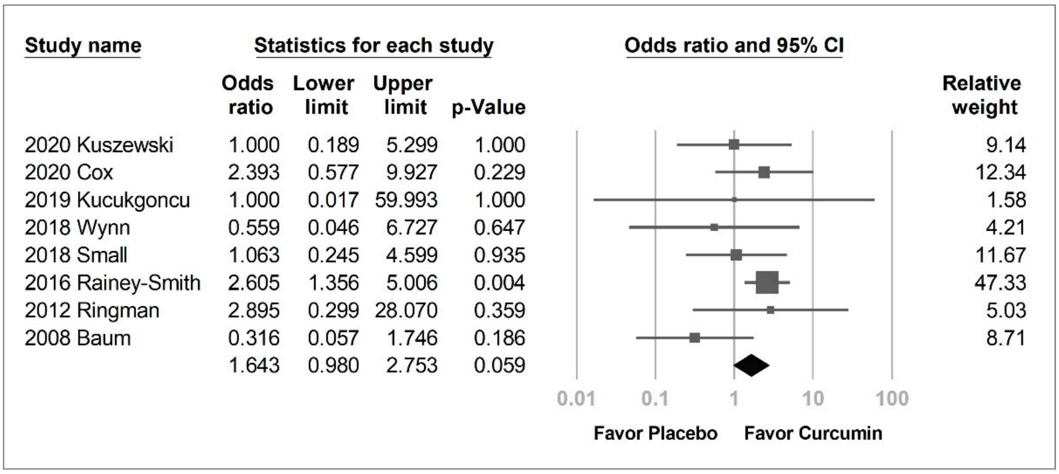

Figure S8. Forest plot of the effect of curcumin on withdrawal rate compared with the placebo (OR = 1.643, 95% CI = 0.980 to 2.753, $p = 0.059$, $I^2 = 5.5\%$)

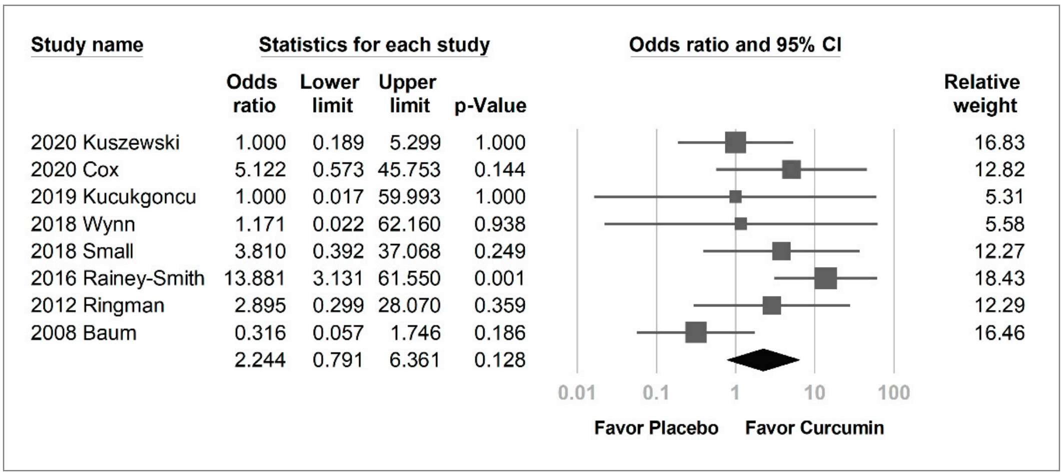

Figure S9. Forest plot of the effect of curcumin on adverse event-related withdrawal rate compared with the placebo (OR = 2.244, 95% CI = 0.791 to 6.361, $p = 0.128$, $I^2 = 45.1\%$)

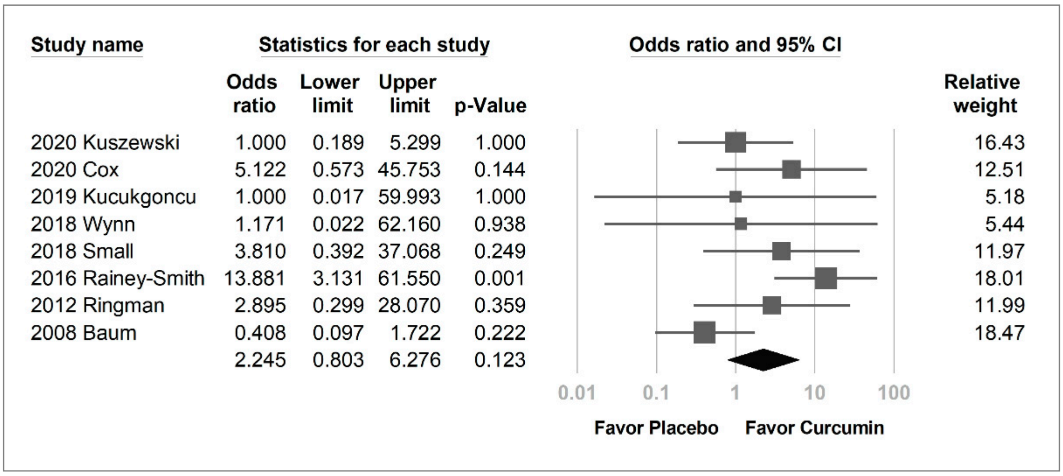

Figure S10. Forest plot of the effect of curcumin on adverse event rate compared with the placebo (OR = 2.245, 95% CI = 0.803 to 6.276, $p = 0.123$, $I^2 = 46.5\%$)

Supplementary Figures / Part 2 – Funnel Plots

The following figures show the funnel plots of the studies evaluating different domains of cognitive function. Since in all domains, the included studies were less than 10, no Egger's test was performed, due to an expectedly low power [1,2]. In the domains with fewer than three studies, the funnel plot cannot be created and is thus not listed below.

1. Page, M.J.; Higgins, J.P.T.; Sterne, J.A.C. Chapter 13: Assessing risk of bias due to missing results in a synthesis. Cochrane Handbook for Systematic Reviews of Interventions. Version 6.2. Available online: https://training.cochrane.org/handbook/current/chapter-13 (accessed on Aug 13, 2021).
2. Egger, M.; Davey Smith, G.; Schneider, M.; Minder, C. Bias in meta-analysis detected by a simple, graphical test. Bmj 1997, 315, 629-634.

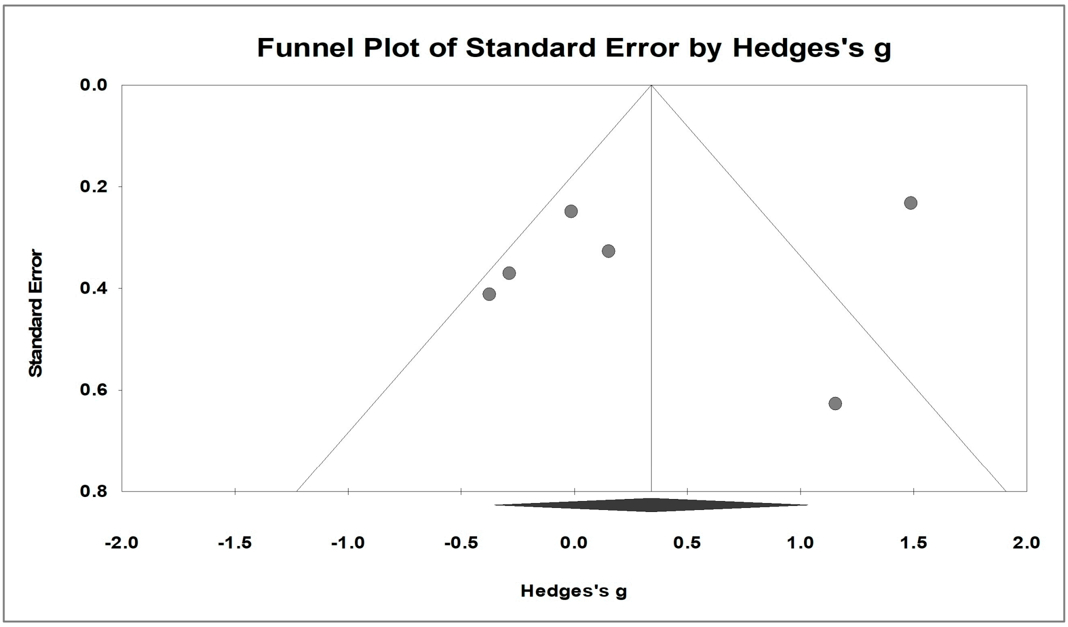

Figure S11. Funnel plot of the studies evaluating overall cognitive performance

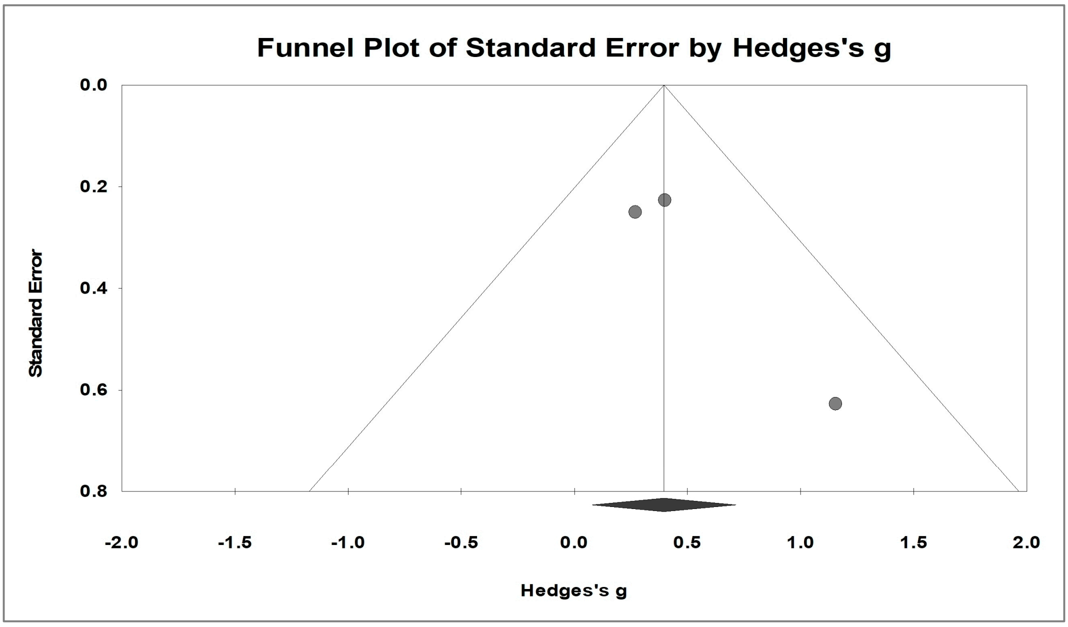

Figure S12. Funnel plot of the studies evaluating working memory

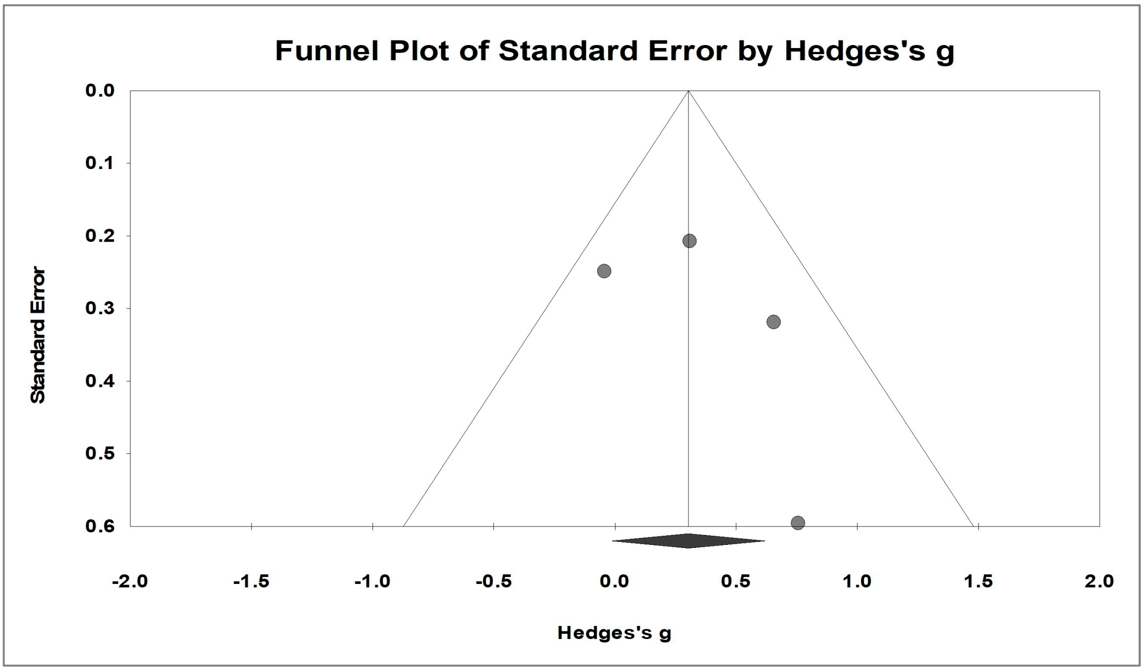

Figure S13. Funnel plot of the studies evaluating processing speed

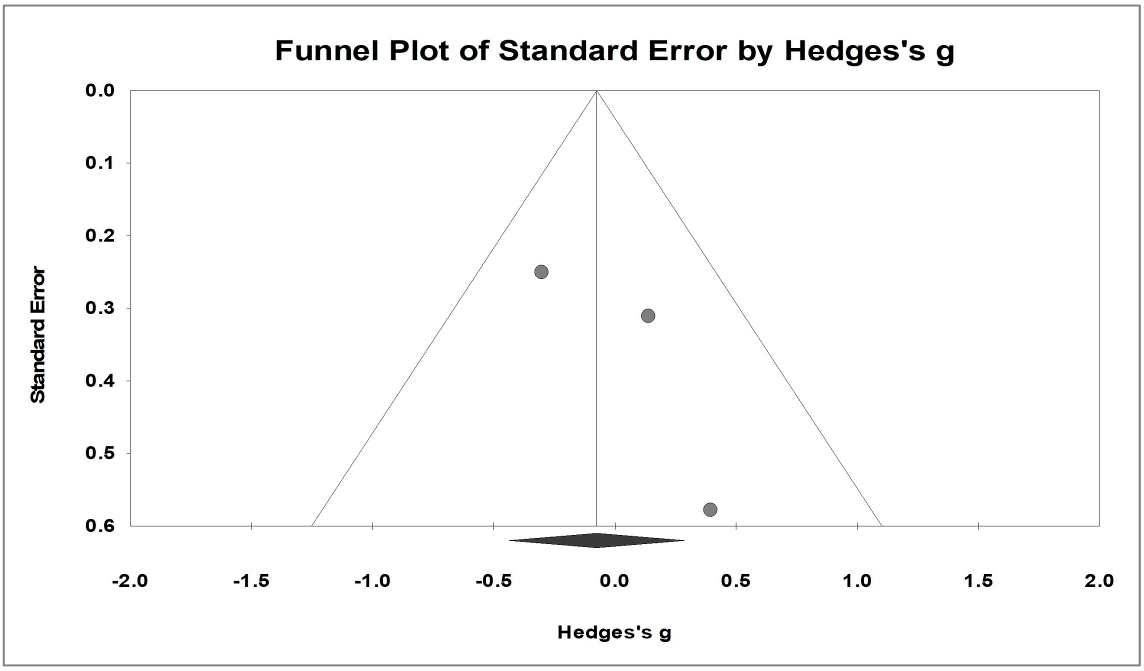

Figure S14. Funnel plot of the studies evaluating episodic memory/visual learning

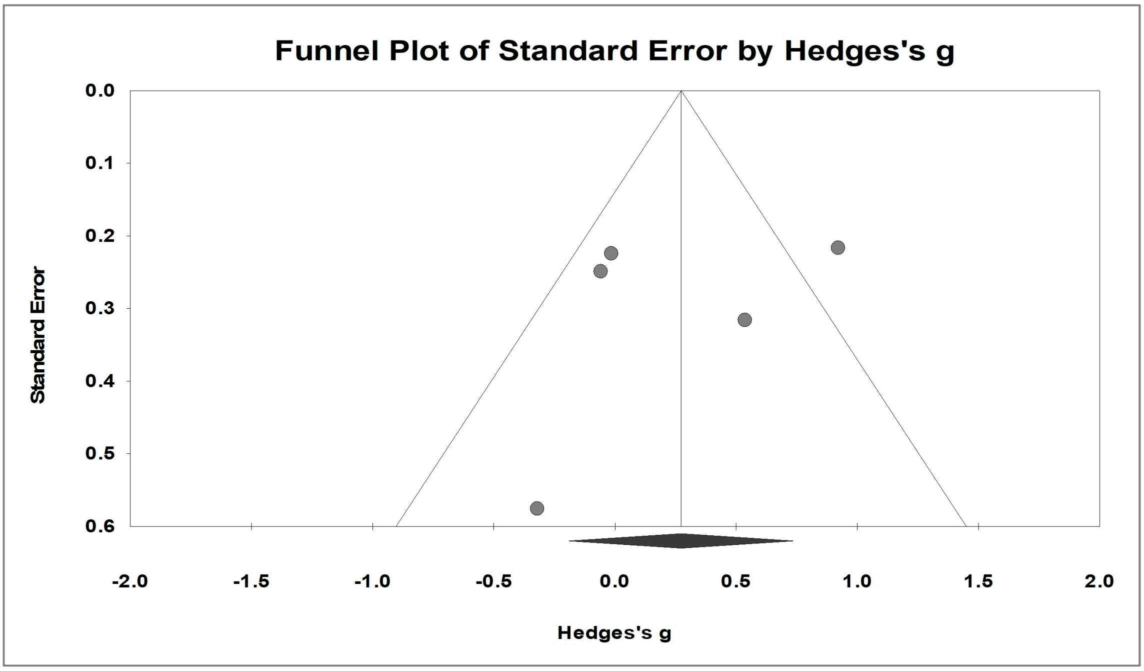

Figure S15. Funnel plot of the studies evaluating verbal memory

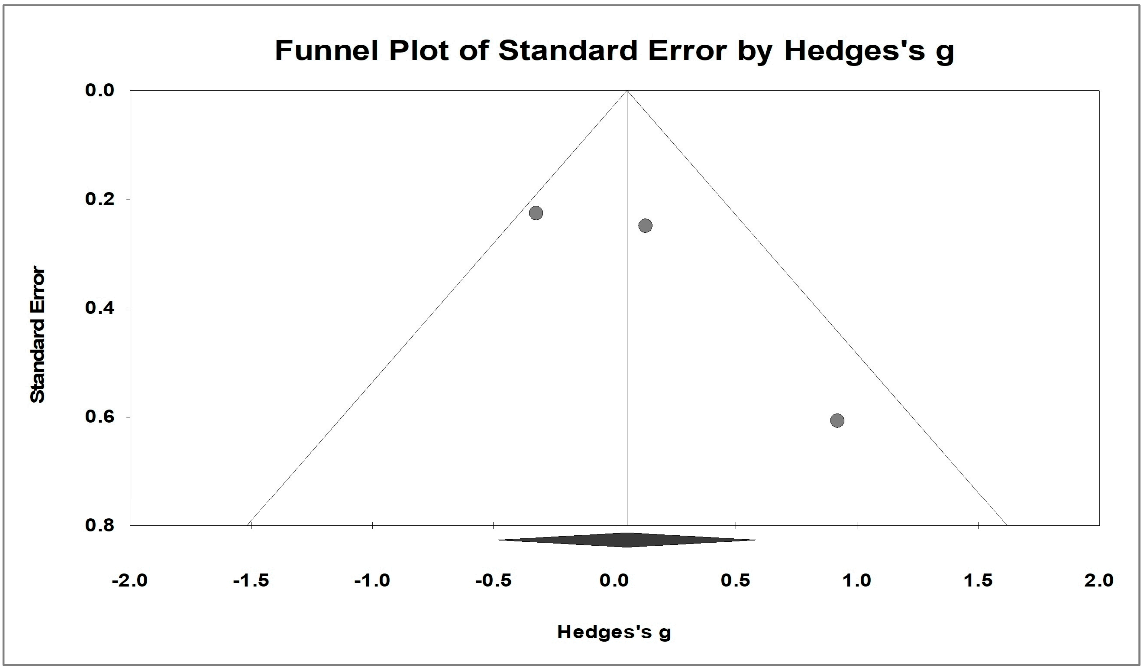

Figure S16. Funnel plot of the studies evaluating cognitive flexibility/problem solving

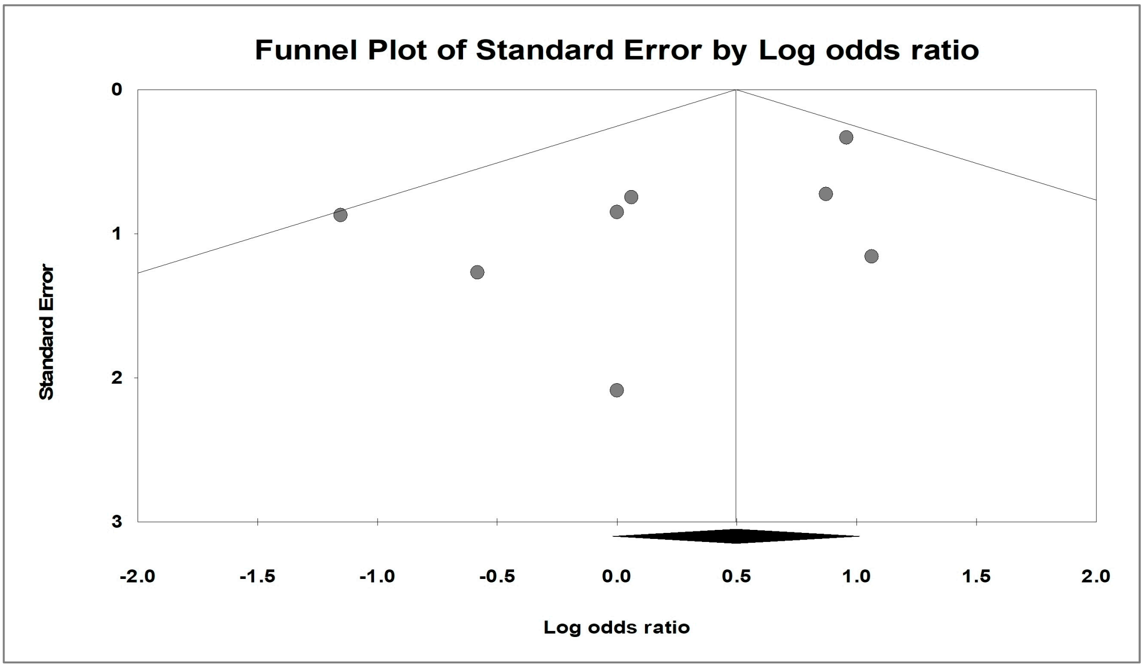

Figure S17. Funnel plot of the studies evaluating withdrawal rate

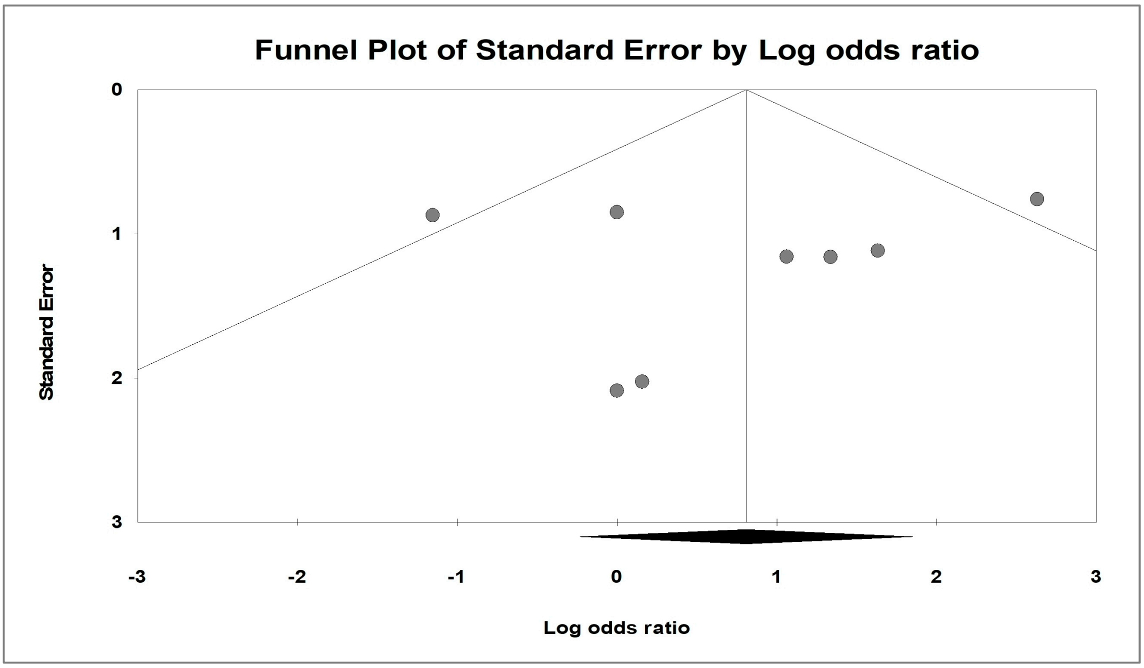

Figure S18. Funnel plot of the studies evaluating the adverse event-related withdrawal rate

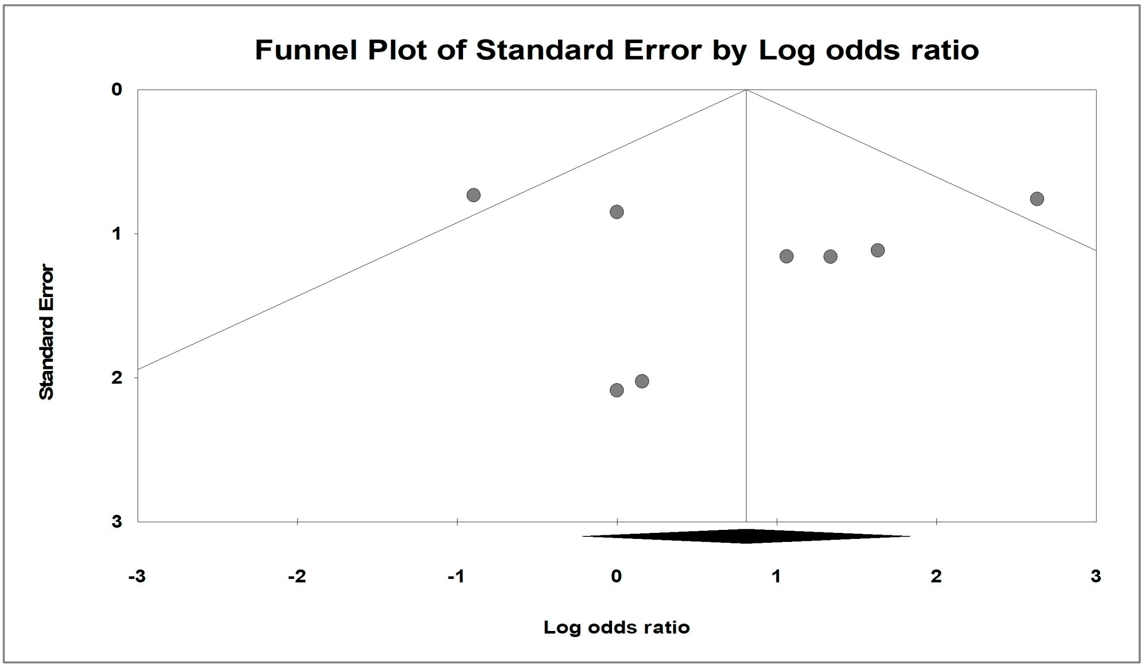

Figure S19. Funnel plot of studies the evaluating the adverse event rate

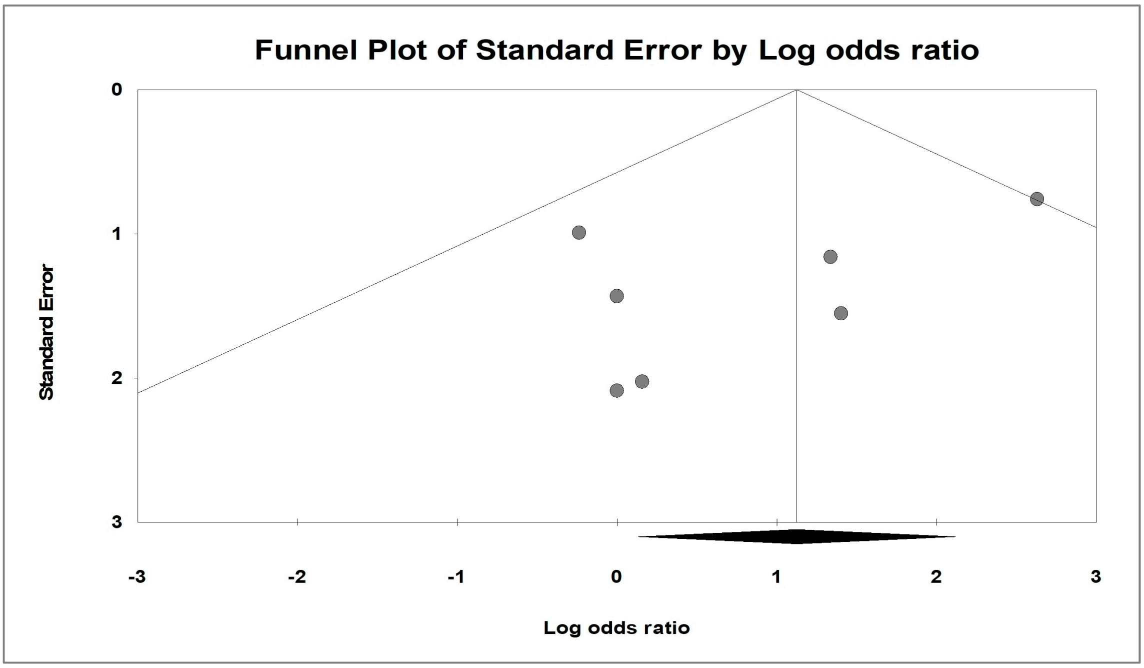

Figure S20. Funnel plot of the studies evaluating the gastrointestinal adverse event rate

Table S1. Excluded studies and reasons

Reasons	Details	Citations
Curcumin intervention less than 8 weeks	Baseline, acute (1 and 3 h after a single dose), chronic (4 weeks) and acute-on-chronic (1 and 3 h after single dose following chronic treatment) effects of solid lipid curcumin formulation on cognitive function	Cox, K. H. M.; Pipingas, A.; Scholey, A. B. Investigation of the Effects of Solid Lipid Curcumin on Cognition and Mood in a Healthy Older Population. J. Psychopharmacol. 2015, 29 (5), 642–651. doi: 10.1177/0269881114552744
Curcumin intervention less than 8 weeks	Pre- and 6-hour post-working memory after single dose curcumin	Lee, M.-S.; Wahlqvist, M. L.; Chou, Y.-C.; Fang, W.-H.; Lee, J.-T.; Kuan, J.-C.; Liu, H.-Y.; Lu, T.-M.; Xiu, L.; Hsu, C.-C.; Andrews, Z. B.; Pan, W.-H. Turmeric Improves Post-Prandial Working Memory in Pre-Diabetes Independent of Insulin. Asia Pac. J. Clin. Nutr. 2014, 23 (4), 581–591. doi: 10.6133/apjcn.2014.23.4.24
Introductory research review with content duplication	This article reported and introduced Small GW et al.'s 2018 study [1], which was already included in the meta-analysis.	Ross, S. M. Curcuma Longa (Theracumin®): A Bioavailable Form of Curcumin and Its Cognitive Benefits. Holist. Nurs. Pract. 2018, 32 (4), 217–220. doi: 10.1097/HNP.0000000000000281

References

1. Small, G. W.; Siddarth, P.; Li, Z.; Miller, K. J.; Ercoli, L.; Emerson, N. D.; Martinez, J.; Wong, K.-P.; Liu, J.; Merrill, D. A.; Chen, S. T.; Henning, S. M.; Satyamurthy, N.; Huang, S.-C.; Heber, D.; Barrio, J. R. Memory and Brain Amyloid and Tau Effects of a Bioavailable Form of Curcumin in Non-Demented Adults: A Double-Blind, Placebo-Controlled 18-Month Trial. Am. J. Geriatr. Psychiatry 2018, 26 (3), 266–277. doi: 10.1016/j.jagp.2017.10.010

Table S2. PRISMA Checklist

Section and Topic	#	Checklist item	Location
**TITLE**			
Title	1	Identify the report as a systematic review.	P1
**ABSTRACT**			
Abstract	2	See the PRISMA 2020 for Abstracts checklist.	P2
**INTRODUCTION**			
Rationale	3	Describe the rationale for the review in the context of existing knowledge.	P3
Objectives	4	Provide an explicit statement of the objective(s) or question(s) the review addresses.	P3
**METHODS**			
Eligibility criteria	5	Specify the inclusion and exclusion criteria for the review and how studies were grouped for the syntheses.	P4
Information sources	6	Specify all databases, registers, websites, organisations, reference lists and other sources searched or consulted to identify studies. Specify the date when each source was last searched or consulted.	P4
Search strategy	7	Present the full search strategies for all databases, registers and websites, including any filters and limits used.	P4, Table S2
Selection process	8	Specify the methods used to decide whether a study met the inclusion criteria of the review, including how many reviewers screened each record and each report retrieved, whether they worked independently, and if applicable, details of automation tools used in the process.	P4
Data collection process	9	Specify the methods used to collect data from reports, including how many reviewers collected data from each report, whether they worked independently, any processes for obtaining or confirming data from study investigators, and if applicable, details of automation tools used in the process.	P5
Data items	10a	List and define all outcomes for which data were sought. Specify whether all results that were compatible with each outcome domain in each study were sought (e.g., for all measures, time points, analyses), and if not, the methods used to decide which results to collect.	P5
	10b	List and define all other variables for which data were sought (e.g., participant and intervention characteristics, funding sources). Describe any assumptions made about any missing or unclear information.	P5, Table 1
Study risk of bias assessment	11	Specify the methods used to assess risk of bias in the included studies, including details of the tool(s) used, how many reviewers assessed each study and whether they worked independently, and if applicable, details of automation tools used in the process.	P4-5
Effect measures	12	Specify for each outcome the effect measure(s) (e.g., risk ratio, mean difference) used in the synthesis or presentation of results.	P5
Synthesis methods	13a	Describe the processes used to decide which studies were eligible for each synthesis (e.g., tabulating the study intervention characteristics and comparing against the planned groups for each synthesis (item #5)).	P5-6, Table 1
	13b	Describe any methods required to prepare the data for presentation or synthesis, such as handling of missing summary statistics, or data conversions.	P5
	13c	Describe any methods used to tabulate or visually display results of individual studies and syntheses.	P5
	13d	Describe any methods used to synthesize results and provide a rationale for the choice(s). If meta-analysis was performed, describe the model(s), method(s) to identify the presence and extent of statistical heterogeneity, and software package(s) used.	P5-6
	13e	Describe any methods used to explore possible causes of heterogeneity among study results (e.g., subgroup analysis, meta-regression).	P6
	13f	Describe any sensitivity analyses conducted to assess robustness of the synthesized results.	P5-6
Reporting bias assessment	14	Describe any methods used to assess risk of bias due to missing results in a synthesis (arising from reporting biases).	Table 2
Certainty assessment	15	Describe any methods used to assess certainty (or confidence) in the body of evidence for an outcome.	P5-6

**RESULTS**			
Study selection	16a	Describe the results of the search and selection process, from the number of records identified in the search to the number of studies included in the review, ideally using a flow diagram.	P7, Figure 1
	16b	Cite studies that might appear to meet the inclusion criteria, but which were excluded, and explain why they were excluded.	P7, Table S3
Study characteristics	17	Cite each included study and present its characteristics.	P7, Table 1
Risk of bias	18	Present assessments of risk of bias for each included study.	Table 2
Results of individual studies	19	For all outcomes, present, for each study: (a) summary statistics for each group (where appropriate) and (b) an effect estimates and its precision (e.g., confidence/credible interval), ideally using structured tables or plots.	Figure 2-6<br>Figure S1-S6
Results of syntheses	20a	For each synthesis, briefly summarise the characteristics and risk of bias among contributing studies.	P7, Table 2
	20b	Present results of all statistical syntheses conducted. If meta-analysis was done, present for each the summary estimate and its precision (e.g., confidence/credible interval) and measures of statistical heterogeneity. If comparing groups, describe the direction of the effect.	Figure 2-6<br>Figure S1-S6
	20c	Present results of all investigations of possible causes of heterogeneity among study results.	P7-9
	20d	Present results of all sensitivity analyses conducted to assess the robustness of the synthesized results.	P8, Figure S1-S2
Reporting biases	21	Present assessments of risk of bias due to missing results (arising from reporting biases) for each synthesis assessed.	Table 2
Certainty of evidence	22	Present assessments of certainty (or confidence) in the body of evidence for each outcome assessed.	P7-8
**DISCUSSION**			
Discussion	23a	Provide a general interpretation of the results in the context of other evidence.	P10
	23b	Discuss any limitations of the evidence included in the review.	P11-12
	23c	Discuss any limitations of the review processes used.	P11-12
	23d	Discuss implications of the results for practice, policy, and future research.	P11-12
**OTHER INFORMATION**			
Registration and protocol	24a	Provide registration information for the review, including register name and registration number, or state that the review was not registered.	P4
	24b	Indicate where the review protocol can be accessed, or state that a protocol was not prepared.	P4
	24c	Describe and explain any amendments to information provided at registration or in the protocol.	P4
Support	25	Describe sources of financial or non-financial support for the review, and the role of the funders or sponsors in the review.	P12
Competing interests	26	Declare any competing interests of review authors.	P12
Availability of data, code and other materials	27	Report which of the following are publicly available and where they can be found: template data collection forms; data extracted from included studies; data used for all analyses; analytic code; any other materials used in the review.	P12

Table S3. Keywords and search results in different databases

Database	Keyword*	Filter	Date	Results
PubMed	('curcumin' OR 'turmeric' OR 'ginger' OR 'curcuma longa' OR 'curcuminoid') AND ('cognitive' OR 'cognition' OR 'memory' OR 'learning' OR 'dementia' OR 'Alzheimer')	NA	2021/8/18	1413
Embase	('curcumin' OR 'turmeric' OR 'ginger' OR 'curcuma longa' OR 'curcuminoid') AND ('cognitive' OR 'cognition' OR 'memory' OR 'learning' OR 'dementia' OR 'Alzheimer')	NA	2021/8/18	3548
ClinicalKey	('curcumin' OR 'turmeric' OR 'ginger' OR 'curcuma longa' OR 'curcuminoid') AND ('cognitive' OR 'cognition' OR 'memory' OR 'learning' OR 'dementia' OR 'Alzheimer')	NA	2021/8/18	5
Cochrane CENTRAL	('curcumin' OR 'turmeric' OR 'ginger' OR 'curcuma longa' OR 'curcuminoid') AND ('cognitive' OR 'cognition' OR 'memory' OR 'learning' OR 'dementia' OR 'Alzheimer')	Title Abstract Keyword	2021/8/18	123
Medline (via OVID)	('curcumin' OR 'turmeric' OR 'ginger' OR 'curcuma longa' OR 'curcuminoid') AND ('cognitive' OR 'cognition' OR 'memory' OR 'learning' OR 'dementia' OR 'Alzheimer')	Keyword	2021/8/18	949
Web of Science	('curcumin' OR 'turmeric' OR 'ginger' OR 'curcuma longa' OR 'curcuminoid') AND ('cognitive' OR 'cognition' OR 'memory' OR 'learning' OR 'dementia' OR 'Alzheimer')	NA	2021/8/18	1909
ClinicalTrials.gov	('curcumin' OR 'turmeric' OR 'ginger' OR 'curcuma longa' OR 'curcuminoid') AND ('cognitive' OR 'cognition' OR 'memory' OR 'learning' OR 'dementia' OR 'Alzheimer')	Condition or disease	2021/8/18	15

NA: not applied
